# Signal and contrast to noise ratio evaluation of fluoroscopic loops for interventional fluoroscope quality control

**DOI:** 10.1002/acm2.12734

**Published:** 2019-10-08

**Authors:** Allen R. Goode, Carl Snyder, Angela Snyder, Patricia Collins, Matthew DeLorenzo, Pei‐Jan Lin

**Affiliations:** ^1^ Department of Radiology and Medical Imaging University of Virginia Health System Charlottesville VA USA; ^2^ Atirix Medical Systems Inc. Minneapolis MN USA; ^3^ Department of Radiology Virginia Commonwealth University Medical Center Richmond VA USA

**Keywords:** CNR, fluoroscopy, quality control, SNR

## Abstract

Modern fluoroscopes pose a challenge for the clinical physicist for annual testing and continued upkeep. These fluoroscopes are critical to providing care to patients for complex interventions, and continue to evolve in automated image quality adjustments. Few tools in software or hardware currently exist to assist the physicist or technologist in gauging fluoroscope constancy or readiness for procedures. Many modalities such as mammography, computed tomography or even magnetic resonance imaging are much more evolved with respect to testing or quality control. In this work we sought to provide simple reproducible tools and methods for spot evaluating or continued quality testing of interventional fluoroscopes.

## 1 INTRODUCTION

Complex fluoroscopically guided interventions (FGIs) have become common in many interventional radiology departments. Quality control (QC) is a necessary and appropriate activity to gauge the readiness of the fluoroscopes used in these procedures. The American College of Radiology (ACR) as well as the American Association of Physicists in Medicine recommend a QC program for these devices to ensure accurate and consistent patient care.^1^ However, currently there is no agreed upon objective metric to be used to ascertain changes made to dose and image quality on the images seen during routine testing, QC, or the For Presentation images employed during clinical use. Additionally, fluoroscopes used in FGIs continue to evolve and become more complicated. The National Electrical Manufacturers Association has addressed some of the complexities of performing annual inspections and image quality adjustments by establishing a standard for medical imaging manufacturers to provide a manual operating mode on fluoroscopes to accomplish these tasks.^2^ In this work, we sought to identify simple and reproducible metrics to be used for both periodic QC and continuous operating levels to ensure that the fluoroscope is ready to be used in FGI procedures, and to gauge changes made to organ programs or dose that could affect image quality.

Signal to noise ratio (SNR) and contrast to noise ratio (CNR) are widely used in other x‐ray imaging modalities such as computed tomography (CT) and mammography as QC standards. In CT, CNR is used as a pass/fail criterion for ACR CT accreditation.^3^ In mammography, some manufacturers use either SNR, CNR, or both to set weekly pass/fail limits to monitor performance of the system, as well as pass/fail criteria as part of the new ACR Digital Mammography Accreditation Program.^4^ These pass/fail standards are helpful to the technologists not only for accreditation purposes but also to remove the ambiguity of modality readiness within the quality program. If SNR or CNR do not meet the minimum threshold, the unit or program being used is deemed unfit for patient use. Tapiovaara et al. identified possible metrics of fluoroscopy QC via SNR and Noise Power Spectra,^5^ and other studies have proposed the use of a Leeds phantom (Leeds Test Objects Ltd, North Yorkshire, UK) and a solid state radiation detector to track air kerma and image quality.^6^ Both of these methods required direct analysis by a medical physicist. Now, with the ubiquitous role of Picture Archiving and Communication System (PACS) and vendor neutral archives in modern interventional radiology departments, it is possible to automate much of the image routing and enable image quality tracking across a fleet of fluoroscopes using a centralized database. The centralized database provides a “digital workbench” whereby phantom images acquired daily by the technologists are processed using automated image analysis. The QC data are immediately available to be reviewed and recorded by the technologist.

We begin an initial endeavor into continuous monitoring of image quality in fluoroscopy, via the use of SNR and CNR as metrics for QC and operational readiness, as part of an ongoing in‐house QC program. Technologists perform daily QC using a customized phantom, and after months of data collection, we begin to investigate pass/fail criteria for SNR and CNR. The benefits of such a program include engagement of the technologists, in concert with the physicist, in the quality program, as well as aiding the staff in operational readiness of the fluoroscope.

## 2 MATERIALS AND METHODS

### 2.1 X‐ray fluoroscopes and phantoms

Quality control and testing was performed in two high‐patient‐volume interventional departments at our institution. The Interventional Radiology (IR) department specializes in peripheral angiography and has five Siemens fluoroscopes (Siemens Healthineers, Forchheim, Germany); one Artis Zeego, two Axiom Artis, and two Artis Q single plane systems. The Interventional Neuroradiology (INR) department has two Siemens Artis Zee Bi‐plane systems, which are predominantly used for neurovascular work. All rooms in both departments have the ability to “Store Fluoro,” which is a feature used to save the last fluoro scene as a series or loop that would otherwise not be captured. To achieve steady state dosimetric parameters (kVp, mA, spectral filter), the stored fluoro loops were only saved after multiple activations of the fluoro pedal, a method often described as “double clutching,” to allow a possible spectral filter or other parameters to change if necessary due to increased or different load via our phantoms. Lastly, all fluoro loops were acquired for 5 s, to acquire enough frames for an average to be determined, as well as to allow the system kV or mA to reach steady state within the loop. Three separate and unique phantoms were used for testing based on availability and ease of use. Each is described in more detail to follow.

**Figure 1:**
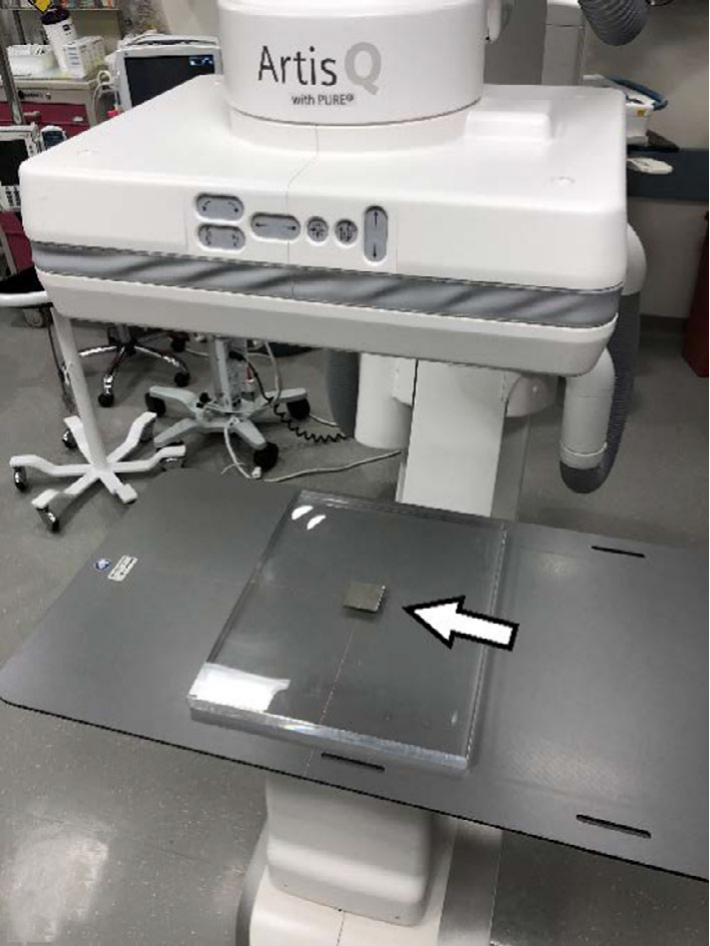
Tin swatch used for contrast to noise ratio measurement directly on top of 1st sheet of polymethyl methacrylate.

**Figure 2:**
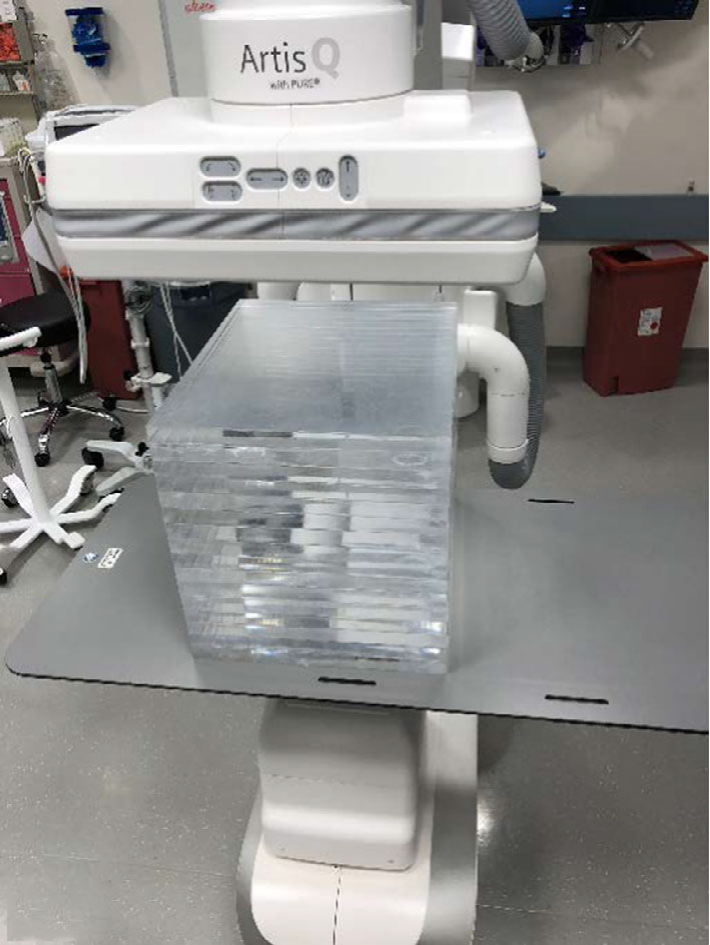
Slab phantom consisting of a stack of 2.54 cm thick 35.5 cm × 43.2 cm polymethyl methacrylate sheets.

The first phantom, called the slab phantom, consisted of 35.5 cm × 43.2 cm × 2.54 cm polymethyl methacrylate (PMMA) sheets that can be stacked at various thicknesses on the patient couch representing varying patient sizes, similar to the method described in TG‐125.^7^ While this phantom was not one singular phantom, collectively the slabs were only used as an initial proof of concept for SNR and CNR testing methodology. A rectangular tin foil swatch (50 mm × 50 mm × 0.05 mm thick) was employed as a target for CNR measurements and utilized as a surrogate for an iodinated contrast agent, similar to the method described by Kotre et al.^8^ The tin swatch (Goodfellow Corporation, Coraopolis, PA, USA) was placed directly on top at the center of the first 2.54 cm slab of PMMA (Fig. 1) and imaged with clinically used programs, at 7.5 pulses per second (pps), at maximum Source To Image Distance (SID), and with the table top at the Interventional Reference Point. Field of View (FOV) was kept constant at 43 cm. Slabs were subsequently added and the resulting phantom was imaged for each added slab, and for each added slab the resulting loop was stored (Fig. 2). At 22.9 cm of PMMA, both low dose and high dose fluoro programs were also tested. Although the table pad is typically in place for patient imaging, and removal would alter the beam characteristics, to ease slab placement and balance, the table pad was removed to provide a flat working surface for initial testing.

**Figure 3:**
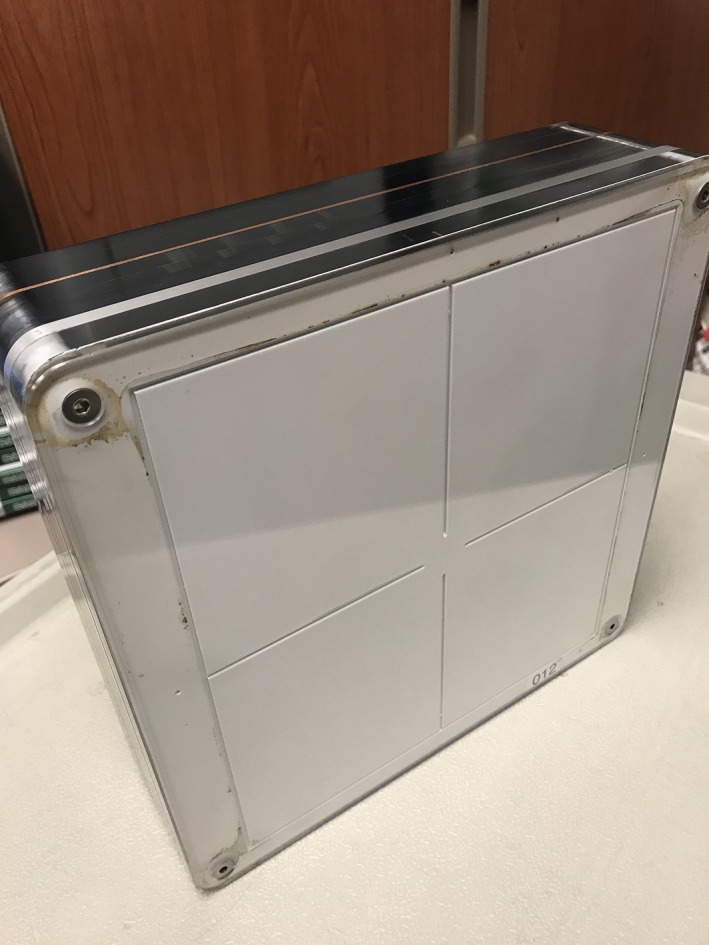
Custom made, patient equivalent QC phantom or “CD Phantom”.

**Figure 4:**
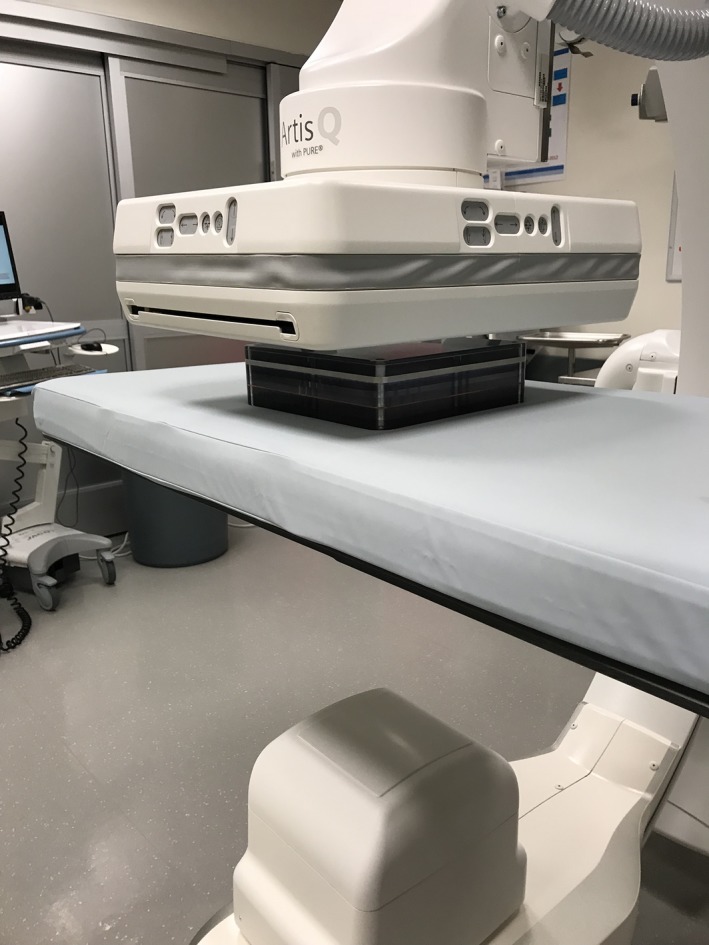
QC phantom in position on patient support.

The second phantom used for testing was a 25.4 cm × 25.4 cm × 7.62 cm custom‐built patient equivalent Contrast‐Detail (CD) phantom consisting of 1.59 mm sheet of copper with a 6.35 mm aluminum sheet sandwiched in additional sheets of PMMA (Fig. 3). The design of this phantom allows for it to be easily carried from room to room. Although this phantom has detail objects inside, there is a space at the center of the phantom, which is free of detail objects and provides a uniform area well suited for SNR measurements. This phantom is currently employed as part of an ongoing daily QC program in our IR department that requires technologists to fluoro the phantom and subjectively count the CD objects or holes. SNR was calculated using the middle, uniform part of this phantom. The phantom was placed on the table pad in the same position each day and centered under fluoroscopy, with a SID set to 100 cm, a nominal FOV set at 32 cm, and with the table raised to a height so that the phantom just met the receptor (Fig. 4). This phantom and setup was used for SNR measurements in all of the IR rooms.

The last phantom, called the CNR phantom, employed in testing closely resembled the CD phantom, except that the detail section containing holes was replaced with uniform polycarbonate and a tin swatch identical to the previously described slab phantom and was centered and affixed within the middle of the phantom. This phantom was used for both SNR and CNR measurements in the INR rooms. A similar setup as described above for the CD phantom was used.

### 2.2 Determination of SNR/CNR from fluoroscopic loops

FGI procedures typically use live fluoroscopy images to assist physicians to perform their tasks. Since live fluoroscopy images are affected by dose per pulse, pulse rate, and frame averaging, we propose that SNR/CNR metrics can and should be performed on fluoroscopic loops as a closer representation of the imaging task used by the fluoroscopist. These loops can be easily stored on the associated fluoroscope workstation or a PACS workstation and analyzed manually or via a custom software solution. Furthermore, in the clinic, iodinated contrast agents are commonly used as positive contrast medium to delineate vessels and techniques to determine blood vessels or flow quantitatively. Individual phantom studies, or serial testing over time on the performance or constancy of visualized contrast, may aid in the visualization of contrast agents clinically.

**Figure 5:**
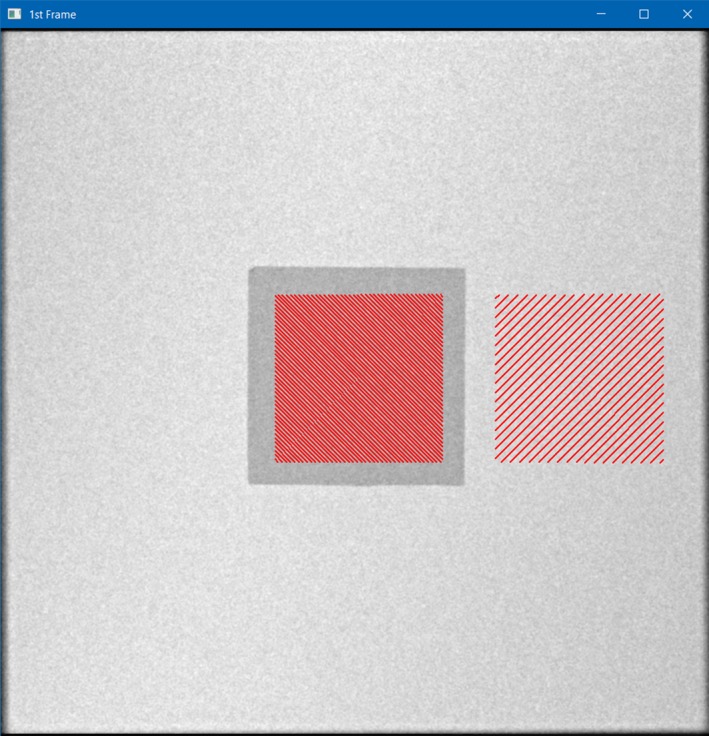
Interactive Data Language screen capture showing 1st Frame of fluoro loop used for ROI selections (red) in center (on Tin swatch) and offset Right (in background) used for signal to noise ratio and Contrast to Noise Ratio measurements using the slab phantom.

Initial testing with the slab phantom was performed in one of the IR rooms. The For Presentation DICOM fluoro loops were exported off‐line to a Personal Computer (PC). A custom program written in IDL (Interactive Data Language, Harris Tech) was used to perform analysis on all frames from the acquired loops. To determine both SNR and CNR, a 40 mm Region of Interest (ROI) was manually placed in the center of the tin swatch. An ROI of the same size was automatically placed with an offset from the tin swatch to provide a background mean value and standard deviation. Figure 5 shows a single frame from one of the fluoro runs. The red hatched areas are the ROIs used for the SNR and CNR measurements. The SNR for each frame was computed via eq. (1):

(1) $${\text{SNR}}_{f}=\frac{{\bar{X}}_{\text{BG}}}{\sigma_{\text{BG}}}$$

where: SNR_f_ is the frame average SNR, ${\bar{X}}_{\text{BG}}$ is the average background signal (pixel value) in a ROI, and $\sigma_{\text{BG}}$ is the standard deviation in the same background ROI.

The CNR for each frame in the loop was determined via eq. (2):

(2) $${\text{CNR}}_{f}=\frac{\left|\bar{S}-{\bar{X}}_{\text{BG}}\right|}{\sigma_{\text{BG}}}$$

where: CNR_f_ is the frame average CNR, ${\bar{X}}_{\text{BG}}$ is the average background signal in an ROI, and $\sigma_{\text{BG}}$ is the standard deviation in the same background ROI and $\bar{S}$ is the mean average signal in the swatch or contrast object.

It was observed that the fluoroscope output rises for the first several frames and eventually stabilizes according to the dose program's automatic dose rate image quality (ADRIQ) logic system settings.^7^ An SNR and CNR for each loop was determined by averaging only the SNR and CNRs for the last 2 s of each loop, in this case 15 frames, to yield a SNR or CNR representative for the loop.

### 2.3 Establishment of daily fluoroscope QC programs

Having established automated analysis of image quality metrics, daily QC was performed for nearly 8 months in the IR department using the previously described CD phantom. In order to provide reproducible and consistent settings, a standard clinical fluoro program was duplicated on each fluoroscope. The program was set to default to 7.5 pulses per second (pps), and named “QC” so it would not be used clinically. The stored For Presentation DICOM loop was sent via standard DICOM networking to a QC‐Track (Atirix Medical Systems, Minneapolis, MN, USA) server where a custom program performed automated SNR analysis, and catalogued the data for each respective fluoroscope. The workflow on the QC‐Track system processed each loop, analyzing only the last two seconds. To reduce the noise, the images were initially smoothed using a Gaussian filter. A Kirsch filter was then applied to find the edges of the contrast holes and an erosion filter was then applied to reduce edge noise. Using a priori knowledge of the contrast hole objects, the largest contrast hole was found using a circle detection algorithm. The 12 mm center ROI was then geometrically placed based from the center of the largest contrast hole. The analysis was repeated for each of the last 15 valid frames of fluoroscopy data, similar to the method used for the slab phantom analysis. The ROI in the center of the phantom was used to determine a frame SNR, and then repeated over all frames and averaged to yield one representative SNR for the entire loop.

**Figure 6:**
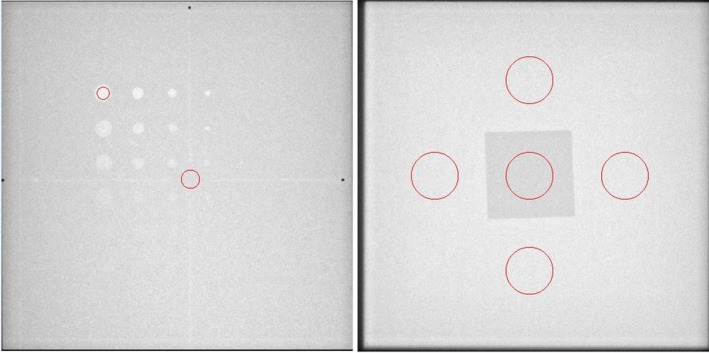
Screen capture from QC‐Track program indicating ROIs used for processing of signal to noise ratio (SNR) on CD phantom (Left) and SNR and contrast to noise ratio (CNR) ROIs from CNR phantom in INR (Right).

Daily QC was also performed over a 5 month period in the INR department using the previously described CNR phantom. As was done in the IR department, the dedicated QC program was built on each fluoroscope. The fluoroscopy loops were acquired in the same way as above with the CD phantom, by technologists pressing the pedal for five seconds using the same setup and dose program. Similar to the SNR measurements, to reduce the noise, the images were initially smoothed using a Gaussian filter. Then a simple thresholding algorithm was used to detect the tin swatch in the center of the phantom. The 30 mm contrast ROI was placed in the centroid of the tin swatch and the 30 mm signal ROIs were geometrically placed around the contrast ROI in the center of the phantom. Data from the ROIs was used to determine a frame CNR by comparing the mean signal value in the central ROI to an averaged signal and standard deviation in the background taken from four ROIs drawn above, below, right, and left of the tin swatch. Multiple background ROIs were used since the detector orientation is not fixed with respect to the phantom. SNR and CNR were then determined for each of the 15 frames and used to yield an average SNR and CNR for each loop. Figure 6 shows the placement of ROIs for a representative frame in a fluoro loop from both phantoms in IR (SNR) and INR (SNR and CNR) respectively.

The circle in the center of the tin swatch (Fig. 6, Right) represents the area used for contrasted signal mean, and the circles at the top bottom, right and left are ROIs used to generate an average background signal and standard deviation to compute SNR and CNR. The mean background signal was determined by averaging the mean signal from the four peripheral ROIs. SNR was computed for each frame by dividing the average peripheral signal by the average peripheral standard deviation.

## 3 RESULTS

### 3.1 SNR‐CNR using slab phantom

**Figure 7:**
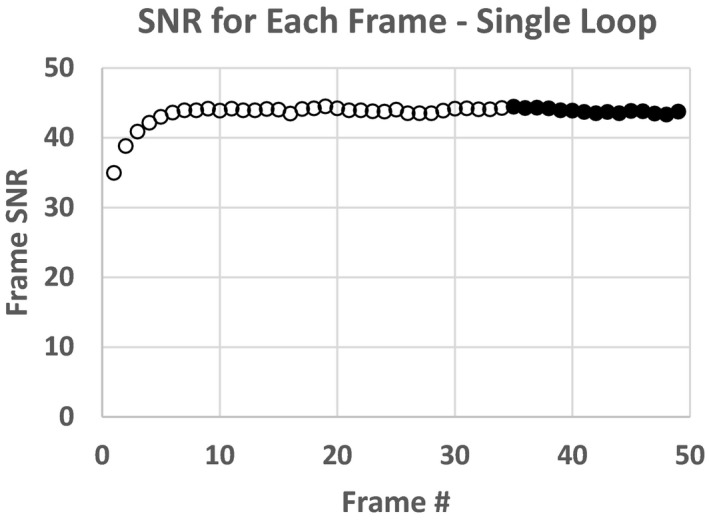
Signal to noise ratio for each frame of fluoro loop for 22.9 cm polymethyl methacrylate slab phantom.

**Figure 8:**
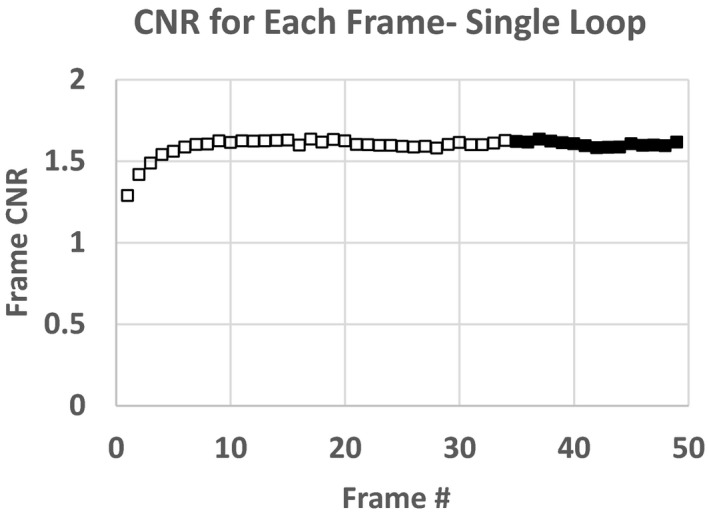
Contrast to noise ratio for each frame of fluoro loop for 22.9 cm polymethyl methacrylate slab phantom.

**Figure 9:**
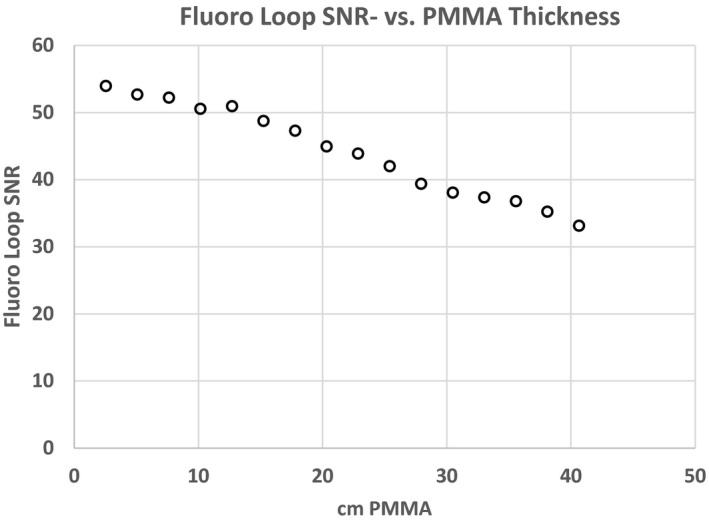
Average signal to noise ratio for each fluoro loop through polymethyl methacrylate slab phantoms of varying thickness.

**Figure 10:**
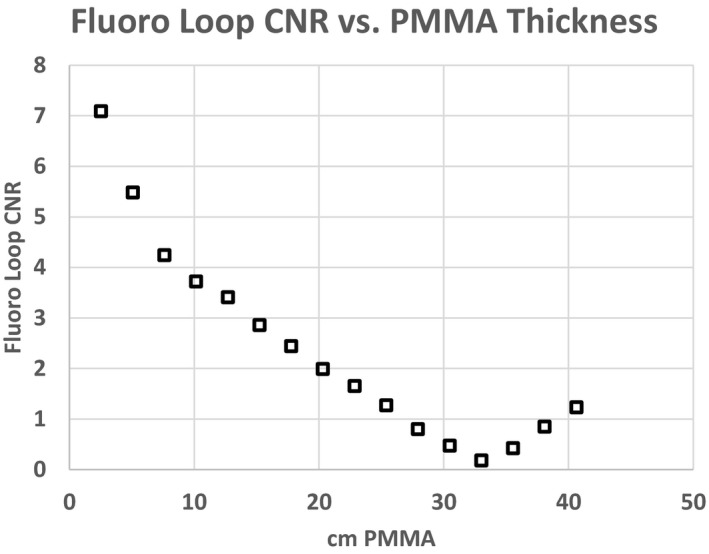
Average contrast to noise ratio (CNR) for each fluoro loop through polymethyl methacrylate slab phantoms of varying thickness. CNR decreases steadily until the phantom thickness reaches 30.5 cm, after which a different beam shaping filter is used by the fluoroscope.

Figures 7 and 8 are plots of the SNR and CNR, respectively, for all frames in the single fluoro loop obtained with 22.96 cm of PMMA. Note the variability in SNR and CNR in the first few frames. Both SNR and CNR appear to stabilize at the end of the acquisition. For this reason, the average SNR or CNR was determined from only the last 2 s of all fluoro loops (solid circle and squares) to give an adequate buffer between the initial variability and steady state conditions. An average SNR or CNR for any single loop was the mean SNR or CNR for the last 15 frames only. The average fluoro loop SNR and CNR were 43.86 and 1.61, respectively. Lastly, Figs. 9 and 10 show the plot of the averaged loop SNR and CNR for each sheet of PMMA, respectively.

### 3.2 Retrospective analysis of daily QC

Figure 12 shows a plot of the daily SNR loop values from one IR room (IR‐19) for nearly 8 months of QC data. The SNR values appear stable and predictable on a daily basis. The kVp chosen by the system for all 158 data sets was 71.3, and the mA consistently ranged from 97 to 99. Using statistical process control logic highlighted by a Shewhart Control Chart, the dashed lines indicate three standard deviations above the mean Upper Control Limit (UCL), while the dash dot lines indicate three standard deviations below the mean Lower Control Limit (LCL).^9^ For the IR room in Fig. 12, only one recording out of 158 (0.6%) fell outside of the control values (LCL at day 40). Coefficient of variation (CV) for the SNR was used as a measure of % error, and ranged from 1.3% (IR‐16) to 3.9% (IR‐18). Table 2 shows a summary of the statistics for all five IR rooms, indicating kVp, mA, SNR, and values above and below the UCL and LCL.

Initially, the daily QC data for CNR for each frame of the fluoro loop were only determined from the ROIs in the center circle (Fig. 6, Right) and background to the right using the CNR phantom. Because a standard detector orientation is not straightforward to specify, multiple background ROIs were used to account for nonuniformities in the detector, which noticeably affect the CNR if only one ROI is used. The background ROI averaging lessened the influence of detector orientation (landscape vs. portrait) on the measurement.

Coefficient of variation (CV) was again used as a measure of % error for both SNR and CNR. Values of CV for the SNR for both units and all planes ranged from a maximum of 3.6% (Neuro Lab‐1, AP) to a minimum of 1.9% for both Neuro Lab‐1 and − 2, Lat and AP planes, respectively. However, in comparison, even though the standard deviations for the SNR were higher than the CNR, the low mean values of the CNR elevated the CV values, and in all cases were more than double the CV of the corresponding SNR CV.

Figure 13 shows both the SNR and CNR daily average loop values for the Neuro Lab‐1 Lat Plane. With the exception of a few SNR values that appear low, the data appear stable and without trend. The values of SNR falling below the average have loop lengths shorter than most and likely did not allow the fluoroscope to adequately stabilize.

## 4 DISCUSSION

In section 3.A, we showed both temporal response, and fidelity of SNR and CNR using a single fluoroscope. In section 3.A Figs. 7 and 8 show that fluoroscopes take several frames to reach steady state and therefore for SNR or CNR to be accurately measured, one must wait until the system has reached quiescence before determining these metrics. The last 2 s of each of the fluoro loops worked well for these tests to strike a balance between premature pedal release (loop too short, averaged over the initial variable phase) verses much longer than 5 s and redundancy of frames post steady state. Since the 5 s loops seemingly provided ample steady state frames — the technologists were instructed to use the same fluoro loop length for the CD and SNR daily QC tests. However, further testing should be performed on differing pulse rates, total number of frames averaged and rise time to steady state to determine the final SNR or CNR.

Section 3.A also showed the relationship between SNR and CNR with respect to a surrogate patient load (PMMA). Figure 9 indicates that even with a large phantom thickness (40.64 cm thickness), the SNR for the system does not appear to decline sharply, but rather decreases slowly. Since the SNR does not abruptly or significantly change, we would anticipate the image quality should follow as the patients get thicker. However, this testing was under one set of conditions, and could be compared to a second set of conditions to determine if there are more optimal settings for differing patient thicknesses.

The CNR results presented in Fig. 10 show a slightly different pattern with respect to SNR. The CNR of the tin iodine surrogate, shows a significant decline with thickness of PMMA, and a complete loss of low contrast resolution at 33 cm. The reason for the increase in CNR at 35.6 cm and greater is both due to a spectral filter change and subsequent change in kVp. This test may provide insight into the fidelity of a particular fluoro program and perhaps where the iodinated contrast agent is most or least effective for a given set of parameters. Again, these settings could be compared to another program for image quality optimization purposes.

**Figure 11:**
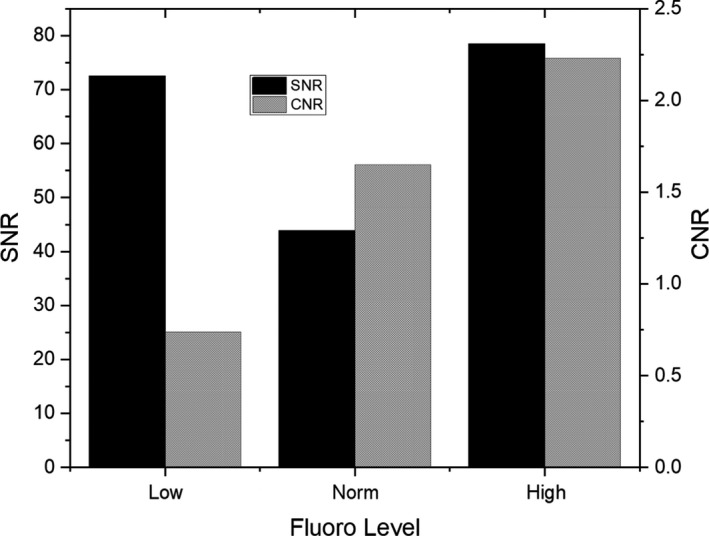
Signal to noise ratio (SNR) and contrast to noise ratio (CNR) using fixed 22.86 cm polymethyl methacrylate (PMMA) slabs with different fluoro dose levels. Note the change in both SNR and CNR for low and high dose fluoro modes where the dose is lower or higher than the normal fluoro dose setting. From the low to norm setting, the dose rate increased 5 mGy/min, however the kVp decreased by 3, and the copper spectral filter decreased 0.6 mm, indicating that the beam quality was significantly higher for the low fluoro setting compared to the normal setting, which reduced the SNR from low to norm at this elevated thickness of PMMA.

Figure 11 shows the relationships between SNR and CNR for three different fluoro programs for one thickness of PMMA. Often clinicians will use the low dose setting, with the intent to lower the dose rate on fluoroscopy. Table 1 shows that for these conditions, and with an increase in kVp and lower dose compared to the normal setting while the SNR is 73 for the low dose program, the CNR is quite low compared with normal and high fluoro settings. Having both SNR and CNR attributes for differing settings on the fluoroscope may aid staff in choice of program selection but also allow the clinical physicist to make changes and compare “A vs. B” and better understand quantitatively the tradeoffs associated with the change. Lastly, the initial testing of the slab phantoms was under optimal conditions and only as proof of concept. Time and care were taken for slab placement as well as removal of the table pad for ease of measurements. Subsequent measurements made by CD and CNR phantoms early in the morning for QC purposes used a more clinical setup. It was felt that for the CD and CNR phantom QC, consistency was key, and even though the support pad altered the X‐ray beam with respect to initial slab phantom testing, haste before starting the patients took priority when moving from the slab phantom to the CD and CNR phantom daily QC and hence, the support pad was left on the table.

**Table 1:** Parameters obtained for constant PMMA SNR/CNR determination. Given the kVp, mA and filtration, as expected the RPAK increases from Low, to Normal to High Fluoro settings.

	Low dose	Normal fluoro	High dose
kVp	74.2	71.3	66.4
mA	125.3	98.7	150.1
Disp RPAK (mGy)	4	9	14
Spectral Filter, Cu (mm)	0.9	0.3	0.3

Abbreviations: SNR, signal to noise ratio; CNR, contrast to noise ratio; PMMA, polymethyl methacrylate; RPAK, reference point air kerma.

**Figure 12:**
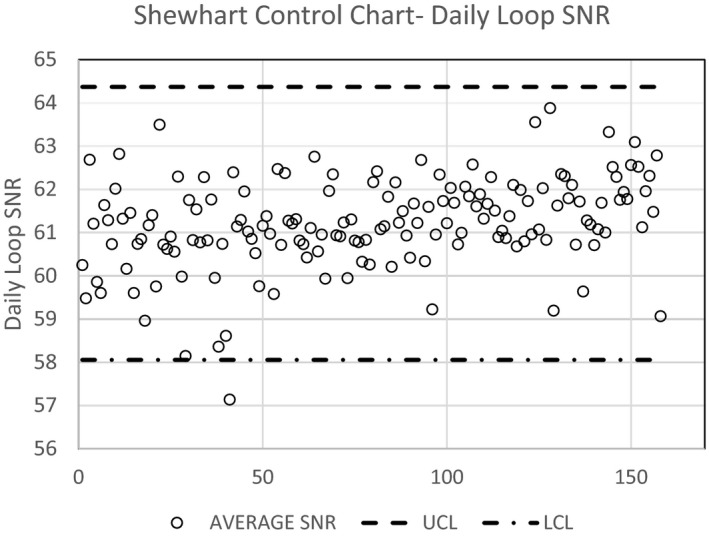
Shewhart Control Chart for Data from IR‐19 using the CD phantom.

Figure 12 shows a Shewhart control chart as an example of one of the IR rooms complete set of data. The significance of the control chart is that, using statistical process control, as in manufacturing, the data should not lie outside three standard deviations from the mean, otherwise the data and related task or result are in error or should be repeated. For IR‐19, only one fluoro loop fell outside the three standard deviations. No attempts were made to put limits or boundaries on the QC data during morning testing. Analysis of the fluoro loops associated with the low SNR outliers indicated one loop with very few frames. In the future, loops with fewer than required frames should be rejected. Other possible causes for the SNR outliers may be attributed to phantom placement and warrant further investigation during future QC. With these results, both upper and lower limits can now be set independently for each room, thereby establishing a threshold when one should repeat the QC, or possibly seek service if the room continues to not be able to be within the boundaries of the daily QC. Table 2 shows the results from the daily QC, and subsequent loop SNR determination. Coefficient of variation for all data for each of the rooms, was less than 4%, and only one fluoroscope (IR‐18) had more than one instance of data above for UCL or below the LCL.

**Table 2:** Fluoroscope loop technique and Shewhart Control Chart Data from all five IR rooms using the CD phantom.

	IR‐15	IR‐16	IR‐17	IR‐18	IR‐19
N days	160	157	154	158	158
Mean kVp	65.7	66.0	67.1	65.9	71.3
kVp SD	1.0	0	1.5	0.28	0
Mean mA	227.3	144.9	175.2	205.9	97.9
mA SD	43.2	1.1	58.3	6.4	0.4
Mean SNR	49.8	50.9	48.1	23.6	61.2
SD SNR	1.21	0.66	1.08	0.92	1.05
CV SNR	2.4%	1.3%	2.2%	3.9%	1.7%
# instances above UCL	1	0	0	0	0
# instances below LCL	0	1	0	3	1

Abbreviations: SNR, signal to noise ratio; CNR, contrast to noise ratio; CV, coefficient of variation; UCL, upper control limit.

**Figure 13:**
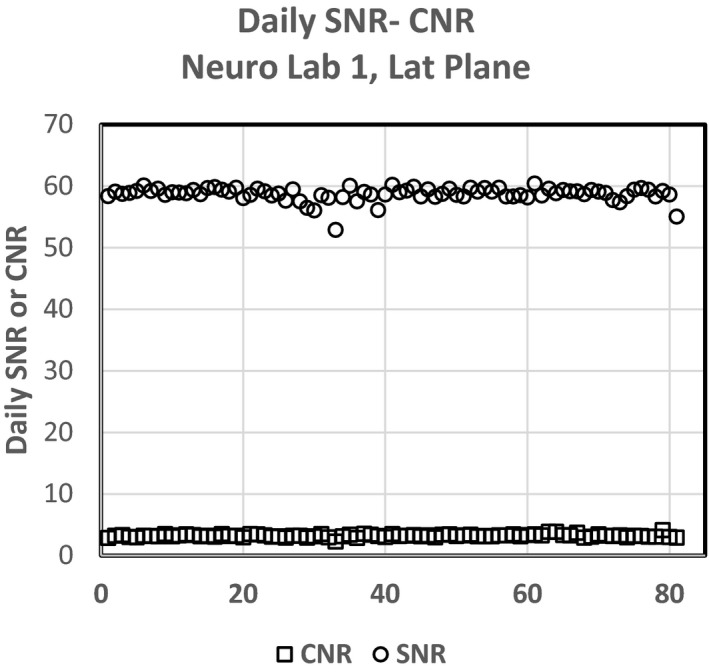
Signal to noise ratio and contrast to noise ratio daily values for Neuro Lab‐ 1, Lat plane.

**Figure 14:**
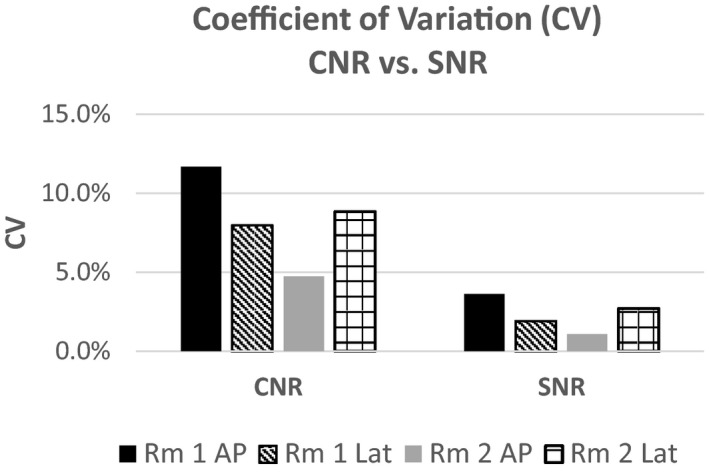
Differences in CV from signal to noise ratio to contrast to noise ratio.

Figure 13 shows a plot from one plane of the INR fluoroscope data (INR‐1, Lat plane) with SNR (circles) and the CNR (squares). The SNR QC data look similar and are without trend with minimal outliers. Table 3 however indicates that the CNR results for each of the fluoroscopes has a large coefficient of variation. Three of the planes of CNR data are approaching 10% error, and one (INR‐1, Lat) is approaching 12% error. For CNR to be used as a daily QC value, further work needs to be performed to determine the source of this elevated error but could possibly be a result of the low contrast value, ROI placement, or something additional. However, for the INR Labs and combined SNR‐CNR evaluation, even though the CNR % errors appear high, the Shewhart control values do not indicate a process control issue with CNR greater than the SNR values. Further work needs to be performed to determine the origin of the elevated error for CNR. Figure 14 shows the comparison of SNR and CNR CV data, and indicates the elevated CV values for CNR vs. SNR for these four fluoroscopes.

**Table 3:** Shows the results from CNR/SNR data for four planes of two biplane fluoroscopes over a 4 month period.

	Neuro Lab‐1				Neuro Lab‐2			
	AP		LAT		AP		LAT	
	CNR	SNR	CNR	SNR	CNR	SNR	CNR	SNR
n	81	81	82	82	77	77	81	81
Mean	3.042	56.54	3.24	58.74	3.24	58.74	2.646	50.37
SD	0.356	2.047	0.252	1.135	0.252	1.135	0.234	1.366
% Error (CV)	11.7%	3.6%	7.8%	1.9%	7.8%	1.9%	8.8%	2.7%
# Above UCL	0	0	1	0	0	0	0	0
# Below LCL	1	1	1	2	1	2	1	2

Abbreviations: SNR, signal to noise ratio; CNR, contrast to noise ratio; CV, coefficient of variation; UCL, upper control limit.

Now that baselines for SNR and possibly CNR have been established, limits can be placed within QC‐Track prompting the technologist to repeat the QC if it fails. Also, QC on the data acquisition itself could also improve the process. For example, if QC loops were acquired with too few frames to allow adequate analysis feedback to the technologist, it could indicate that the test needs to be repeated. Furthermore, sensitivity of the SNR/CNR measurements needs to be more thoroughly investigated with respect to clinical decisions to fail a fluoroscope. As a simple check of our three standard deviation criterion robustness, we removed the anti‐scatter grid prior to performing QC to simulate a nonready fluoroscope. The CNR value for this test fell below three standard deviations, however the SNR was within three standard deviations of the mean. In this singular simulation, the mA value for the loop dropped 2.4 % from the mean, indicating that there may be a need to programmatically examine multiple DICOM header values (mA, kV, DAP) as well as calculated SNR/CNR before making a QC failure decision.

The limitations of this study are that there was only one fluoroscope vendor available for these tests, and a small sample of models from that vendor. Future work will include at least two more vendors and several more models to fully understand SNR and CNR utility and clinical impact in fluoroscopy. Automated and observer‐independent QC of units used during FGIs was performed in high‐patient volume IR and INR departments. Minimal technologist effort and change in workflow were needed to regularly monitor system performance and readiness of the system for the day. These data allow for room‐specific SNR thresholds to be established and used as a criterion for providing immediate feedback on whether the system is operating at an expected level.

## 5 CONCLUSIONS

With the ever increasing complexity of fluoroscopes, coupled with proprietary dose and image quality software algorithms from the vendors, robust and straightforward methods for QC and image quality metrics are needed. We have shown the utility in simple SNR and CNR metrics and how they can be used during fluoroscopy daily QC or during performance testing with very simple patient equivalent phantoms.

## CONFLICT OF INTEREST

The authors have no relevant conflict of interest.
